# Involvement of sigma-1 receptor in astrocyte activation induced by methamphetamine via up-regulation of its own expression

**DOI:** 10.1186/s12974-015-0250-7

**Published:** 2015-02-17

**Authors:** Yuan Zhang, Xuan Lv, Ying Bai, Xinjian Zhu, Xiaodong Wu, Jie Chao, Ming Duan, Shilpa Buch, Ling Chen, Honghong Yao

**Affiliations:** Department of Pharmacology, Medical School of Southeast University, 87 Dingjiaqiao, Nanjing, Jiangsu 210009 China; Department of Physiology, Medical School of Southeast University, 87 Dingjiaqiao, Nanjing, 210009 China; Virosis Laboratory, Key Laboratory of Zoonosis, Ministry of Education, Institute of Zoonosis, Jilin University, 5333 Xi An Road, Changchun, 130062 China; Department of Pharmacology and Experimental Neuroscience, University of Nebraska Medical Center, 42nd and Emile, Omaha, NE 68198 USA; Department of Physiology, Nanjing Medical University, 140 Hanzhong Road, Nanjing, 210029 China

**Keywords:** Sigma-1 receptor, Methamphetamine, Astrocytes, ERK, CREB

## Abstract

**Background:**

Although it has been documented that methamphetamine induces astrocyte activation, the mechanism(s) underlying this effect remain poorly understood. We thus sought to examine the molecular mechanisms involved in methamphetamine-mediated activation of astrocytes with a focus on the role of sigma-1 receptor (σ-1R) in this process.

**Methods:**

The expression of σ-1R and glial fibrillary acidic protein (GFAP) was examined by reverse transcription PCR (RT-PCR), real-time PCR, Western blot, and immunofluorescent staining; phosphorylation of cell signaling pathways was detected by Western blot analysis. Immunoprecipitation was used to determine the interaction between σ-1R and p-Src. Chromatin immunoprecipitation (ChIP) assay was employed to discern the binding of cAMP-response element-binding protein (CREB) with the promoter of σ-1R. The role of σ-1R in astrocyte activation was further validated in σ-1R knockout (KO) mice by Western blot combined with immunofluorescent staining.

**Results:**

Exposure of primary rat astrocytes to methamphetamine increased the expression of σ-1R via the activation of Src, ERK mitogen-activated protein kinase, and downstream CREB pathways. Subsequently, CREB translocated into nucleus and interacted with the promoter of σ-1R resulting in increased expression of σ-1R with a concomitant increase in expression of GFAP. This effect was inhibited in cells treated with the σ-1R antagonist-BD1047, thereby implicating the role of σ-1R in the activation of astrocytes. *In vivo* relevance of these findings was further corroborated in σ-1R KO mice that were administered methamphetamine. In the methamphetamine administered mice, there was a failure of the drug to induce activation of astrocytes, an effect that was evident in wild-type (WT) mice exposed to methamphetamine.

**Conclusions:**

The study presented herein demonstrates that methamphetamine-mediated activation of astrocytes involved up-regulation of σ-1R through a positive-feedback mechanism. Understanding the regulation of σ-1R expression could provide insights into the development of potential therapeutic strategies for astrocyte activation induced by methamphetamine.

## Background

Methamphetamine is an addictive pharmacological psychostimulant of the central nervous system (CNS) and is one of the most commonly abused agents by illicit-drug users [1,2]. In addition to its immediate stimulant effects such as euphoria and enhanced energy, methamphetamine use also manifests clinical psychiatric symptoms characterized by cognitive deficits, depression, anxiety, psychotic symptom, and motor deficits [2]. These psychiatric symptoms are associated with neurodegenerative effects of methamphetamine. Although extensive research has focused on development of pharmacological agents that inhibit methamphetamine-induced neurotoxicity, FAD approved pharmacotherapies for treatment of negative effects of methamphetamine are still lacking. Novel approaches aimed at overcoming the negative effects of methamphetamine while are urgently needed in the field.

Methamphetamine is a psychostimulant that is known to exhibit moderate affinity for sigma-1 receptors (σ-1R) expressed in most neuronal cells [3]. σ-1R are unique drug-binding proteins that are present in the CNS as well as in the periphery [4]. Since methamphetamine binding to σ-1R has been known to exert neurotoxicity, the role of σ-1R in methamphetamine-induced immune alteration has been the focus of many studies. For example, σ-1R antagonists such as MS377 and BMY 14802 have been shown to prevent the development of behavioral sensitization to methamphetamine [5,6]. Recent study has also indicated that SN79 (σ-1R antagonist) attenuated astrogliosis induced by methamphetamine [7]. Despite extensive studies, genetic evidence elucidating the role of σ-1R in methamphetamine-induced neurotoxicity is still lacking in the field. Therefore, studies using genetic approaches like σ-1R knockout (KO) animal model are therefore necessary to further dissect the mechanism(s) and role of σ-1R signaling in methamphetamine-associated neuroinflammation.

While the effects of methamphetamine can primarily be attributed to its action on dopamine receptors and transporters, there exists a close relationship between methamphetamine-induced degeneration of dopaminergic terminals in the striatum and astrocyte activation. Astrocytes are the most abundant cell type within the CNS and may play diverse role in regulating and maintaining CNS homeostasis [8,9]. In addition to their physiological function, pathologically, activated astrocytes were characterized by abnormal morphology with excessive proliferation [10-14]. Previous studies have demonstrated the presence of astrocyte activation in the striatum of methamphetamine-treated mice and rat *in vivo* [7,15], as well as *in vitro* [16,17]. It has also been reported that repeated administration or self-administration of methamphetamine induced the expression of σ-1R protein and mRNA [18,19], however, the detailed molecular mechanism(s) underlying this process are elusive. Based on these findings, we hypothesized that methamphetamine activates astrocytes through positive-feedback mechanism(s) via up-regulating the expression of σ-1R.

In the current study, we provide direct evidence that methamphetamine induces astrocyte activation contributing thereby to neuroinflammation in drug abusers via a previously unidentified positive-feedback regulation of σ-1R expression.

## Methods

### Animals

Male C57BL/6 N mice (weighting 20 to 25 g) were purchased from the Model Animal Research Center of Nanjing University (Nanjing, China). σ-1R KO mice obtained from the Laboratory Animal Center of Nanjing Medical University (Nanjing, China) had been backcrossed ten generations to a C57BL/6 N inbred background. All of the animals were housed under conditions of constant temperature (22°C ± 1°C) and humidity on a 12-h light/12-h dark cycle (lights on between 8:30 and 20:30) with free access to food and water. After habituation, animals were divided into two groups and injected with either saline or methamphetamine (30 mg/kg) with a dosing schedule (injected intraperitoneally every 2 hours for a total of four times/day). All animal procedures were performed in strict accordance with the ARRIVE guidelines and animal protocols approved by the Institutional Animal Care and Use Committee of the Medical School of Southeast University.

### Reagents

Methamphetamine was purchased from the National Institute for the Control of Pharmaceutical and Biological Products (Beijing, China). The specific Src kinase inhibitor PP2 and its inactive analog PP3, MEK1/2 inhibitor U0126, were purchased from Calbiochem (San Diego, CA, USA). H-89 was obtained from Selleck (Houston, TX, USA). The concentrations of these inhibitors were based on the concentration-curve study and our previous reports [20].

### Isolation, differentiation, and characterization of primary rat astrocytes

Postnatal (P2 to P3) Sprague-Dawley (SD) rats were obtained from the Laboratory Animal Center of Nanjing Medical University (Nanjing, China). Whole brains of SD rats were dissected and mechanically dissociated using gauze to remove the membranes and large blood vessels. Brain tissues were digested by Trypsin-EDTA (GIBCO, Grand Island, NY, USA) following which the cells were plated on poly-L-lysine pre-coated cell culture flasks in Dulbecco’s modified Eagle’s medium (DMEM) supplemented with FBS (10% *v*/*v*) and penicillin/streptomycin (1% *v*/*v*). Cultures were maintained in a humidified chamber (37°C, 5% CO_2_ incubator). After 7 to 10 days, the astrocytes were harvested by trypsinization.

### Immunocytochemistry

For immunochemistry, the cells were fixed with 4% paraformaldehyde, permeabilized with 0.3% Triton X-100 in phosphate-buffered saline (PBS) and blocked with 10% normal goat serum (NGS) in 0.3% Triton X-100. The cells were incubated with rabbit anti-σ-1R (Invitrogen, Carlsbad, USA; 1:250), rabbit anti-p-Src (Cell Signaling, Danvers, MA, USA; 1:500), or mouse anti-glial fibrillary acidic protein (GFAP) (Sigma-Aldrich, St. Louis, MO, USA; 1:800) antibodies overnight at 4°C. Following washing three times with PBS, the cells were then incubated with AlexaFluor 488-conjugated anti-rabbit IgG or AlexaFluor 594 goat anti-mouse IgG (Invitrogen, Carlsbad, USA; 1:250) for 1 h to detect σ-1R, p-Src, and GFAP. After a final washing with PBS, the slides were mounted with ProLong Gold Anti-fade reagent to visualize the nuclei onto glass slides (Invitrogen, Carlsbad, USA). Immunofluorescence images were captured using confocal microscopy (Olympus FV100, Olympus, Tokyo, Japan). Average intensities of GFAP were calculated using Image J by sampling a 28 × 28 pixel area, 36 images taken from six consecutive sections. Values were reported as average intensity above background ± SD.

### Western blotting (WB)

Cells were treated and lysed with either Mammalian Cell Lysis Kit (Sigma-Aldrich, St. Louis, MO, USA) or NE-PER Nuclear and Cytoplasmic Extraction Kit (Pierce, Rockford, IL, USA). Protein concentrations were determined using the BCA Protein Assay Kit (Pierce, Rockford, IL, USA), and equal amounts of the proteins were electrophoresed in a sodium dodecyl sulfate-polyacrylamide gel (12%) under reducing conditions followed by transfer to PVDF membranes. Blots were blocked with 5% non-fat dry milk in Tris-buffered saline with Tween-20 (TBST). Western blots were then probed with antibodies recognizing p-Src/Src, p-ERK/ERK, cAMP-response element-binding protein (CREB), Histone H3, GAPDH (Cell Signaling, Danvers, MA, USA; 1:1,000), GFAP (Sigma-Aldrich, St. Louis, MO, USA; 1:1,000), σ-1R (Invitrogen, Carlsbad, USA; 1:500), or β-actin (Sigma, St. Louis, MO, USA; 1:4,000). The secondary antibodies were horseradish peroxidase conjugated to goat anti-mouse/rabbit IgG (1:2,000). Signals were detected by chemiluminescence and imaged on the Microchemi 4.2® (DNR, Israel) digital image scanner. Quantification was performed by densitometry using Image J software (NIH).

### Reverse transcription PCR and real-time PCR

Total RNA was extracted with Trizol reagent (Invitrogen, Carlsbad, USA) according to the manufacturer’s instructions. RNA concentration and purity were assessed by OneDrop OD-1000+ spectrophotometer (OneDrop, Nanjing, China). cDNA was generated from mRNA by reverse transcription using PrimeScript RT Master Mix Kit (TaKaRa, Japan). The sequences for the amplification of rat σ-1R were as follows: 5′-GGGTGTTATTCCGTCTAC-3′ (forward) and 5′-GTATAGAAGAGGGTGAGGA-3′ (reverse). The primer sequences for the amplification of rat β-actin were as follows: 5′-CTAGCACCATGAAGATCAAGAT-3′ (forward) and 5′-CCAGGATAGAGCCACCAA-3′ (reverse). For reverse transcription PCR (RT-PCR), the mixtures were annealed at 50°C (1 min), extended at 72 (2 min), and denatured at 94°C (1 min) for 35 cycles. The PCR product was visualized by electrophoresis on 1.2% agarose gels. Real-time PCR was performed using SYBR Green Master Mix (SABiosciences, Frederick, USA) on an Applied Biosystems 7500 Fast Real-Time PCR System (Foster City, USA). Data were normalized using Ct values for β-actin in each sample. To calculate relative mRNA amounts, the average Ct values were subtracted from β-actin values for each target gene to provide changes in Ct value.

### Immunoprecipitation

Astrocytes were collected in RIPA lysis buffer (Beyotime, Nantong, China), and the protein concentrations were determined using the BCA Protein Assay Kit (Pierce, Rockford, IL). Equal amounts of the proteins were incubated with p-Src (Cell Signaling, Danvers, MA, USA; 1:100) antibody overnight at 4°C followed by incubation with 20 μl of protein A sepharose for 90 min at 4°C. The mixture was centrifuged (12,000 rpm, 1 min, 4°C), and the cell pellets were rinsed twice with RIPA lysis buffer. The cell pellets were boiled with 5× Western blot loading buffer and RIPA lysis buffer for 5 min. After spinning (12,000 rpm, 1 min), the supernatants were subjected to Western blot for detection of σ-1R.

### Chromatin immunoprecipitation assay

Chromatin immunoprecipitation (ChIP) assay was performed according to the manufacturer’s instructions (Millipore, Temecula, USA). Fresh formaldehyde (18.5%) was added directly into the medium to crosslink, and the final concentration of formaldehyde is 1%. After incubation for 10 min at room temperature, the unreacted formaldehyde was quenched with 10× Glycine for 5 min at room temperature. Washed cells (cold 1× PBS, twice) were scraped with cold PBS containing 1× protease inhibitor cocktail and then centrifuged (800 × *g*, 5 min, 4°C) to pellet the cells. Nuclei were harvested from cell pellet by lysis buffer containing 1× protease inhibitor cocktail. DNA was sheared by sonication. The sheared cross-linked chromatin was then diluted with dilution buffer, mixed with protein A magnetic beads and corresponding antibodies including CREB, Histone, and IgG, followed by overnight incubation at 4°C. Following washes with a series of cold wash buffers from low salt buffer, high salt buffer to LiCl buffer, and finally with TE buffer, the cross-linked protein/DNA complexes were reversed to free the bound DNA with elution buffer and purified using DNA purification spin columns following the manufacturer’s instructions. Finally, the purified DNA was amplified via PCR to identify promoter region containing CREB binding site “TGACGGAA”. The sequence of the primers used to identify the σ-1R bound by transcription factor CREB was as follows:

sense: 5′-AGTGGTTGAGGCTGAGAGGA-3′,

anti-sense: 5′-TGGAGGATGAAAGGTTGAGG-3′.

### Statistical analysis

Statistical analysis was performed using SigmaPlot software (SigmaPlot 11.0, Systot,. Inc). Data were presented as mean ± SD. Significance of differences between control and samples treated with various drugs was determined by one-way ANOVA, with the Tukey and Bonferroni correction for multiple comparisons. Values of *P* < 0.05 were taken as statistically significant.

## Results

### Methamphetamine-mediated up-regulation of σ-1R in primary rat astrocytes

**Figure 1:**
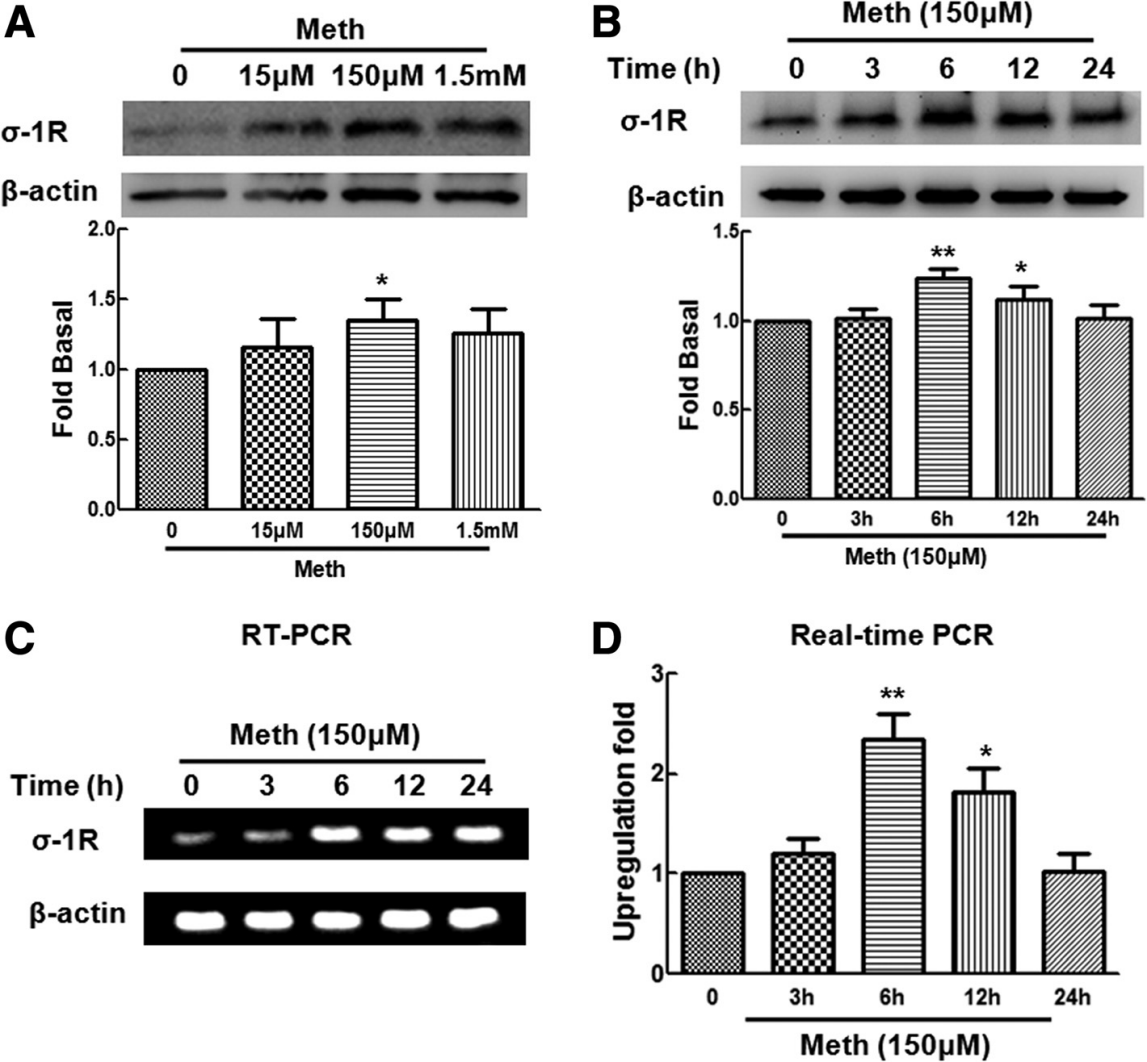
**Methamphetamine-mediated up-regulation of σ-1R in primary rat astrocytes. (A)** Methamphetamine-mediated induction of σ-1R expression in primary rat astrocytes. Cells were incubated with varying concentrations of methamphetamine (15 μM, 150 μM, and 1.5 mM) for 12 h, followed by collection of protein for assay of σ-1R expression by WB. Representative immunoblots and the densitometric analysis of σ-1R/GAPDH from the three separate experiments are presented. **(B)** Methamphetamine induced σ-1R expression in a time-dependent manner in primary rat astrocytes**.** Representative immunoblots and the densitometric analysis of σ-1R/GAPDH from the three separate experiments are presented. **(C, D)** Effect of methamphetamine on σ-1R RNA in primary rat astrocytes. Total RNA isolated from primary rat astrocytes was subjected to RT-PCR (C) and real-time PCR (D) analyses using primer set specific for σ-1R. All the data are presented as mean ± SD of three individual experiments. *P < 0.05 and **P < 0.01 versus control group.

A recent study implicated the role of sigma-1R in methamphetamine-mediated astrogliosis [7]. However, very little is known about the effect of methamphetamine on the expression of σ-1R in astrocytes. To assess the effect of methamphetamine on expression of σ-1R in astrocytes, primary rat astrocytes were exposed to different concentrations of methamphetamine (15 μM, 150 μM, and 1.5 mM) and assessed for the expression of σ-1R. As shown in Figure 1A, methamphetamine induced the expression of σ-1R with peak response at a concentration of 150 μM. To assess the time course of methamphetamine action, we exposed primary rat astrocytes to methamphetamine at 150 μM for varying time points. Compared with the control group, σ-1R was significantly up-regulated at 6 h as shown in Figure 1B.

Having determined that methamphetamine increased the expression of σ-1R at the protein level, the next step was to confirm the effect of methamphetamine on the mRNA level of σ-1R by using RT-PCR as well as real-time PCR assays. As shown in Figure 1C,D, methamphetamine treatment resulted in increased σ-1R RNA in primary astrocytes with a peak response at 6 h. Taken together, these findings clearly demonstrated that methamphetamine treatment increased the expression of σ-1R RNA and protein in primary astrocytes.

### Involvement of σ-1R in methamphetamine-increased σ-1R expression in primary rat astrocytes

**Figure 2:**
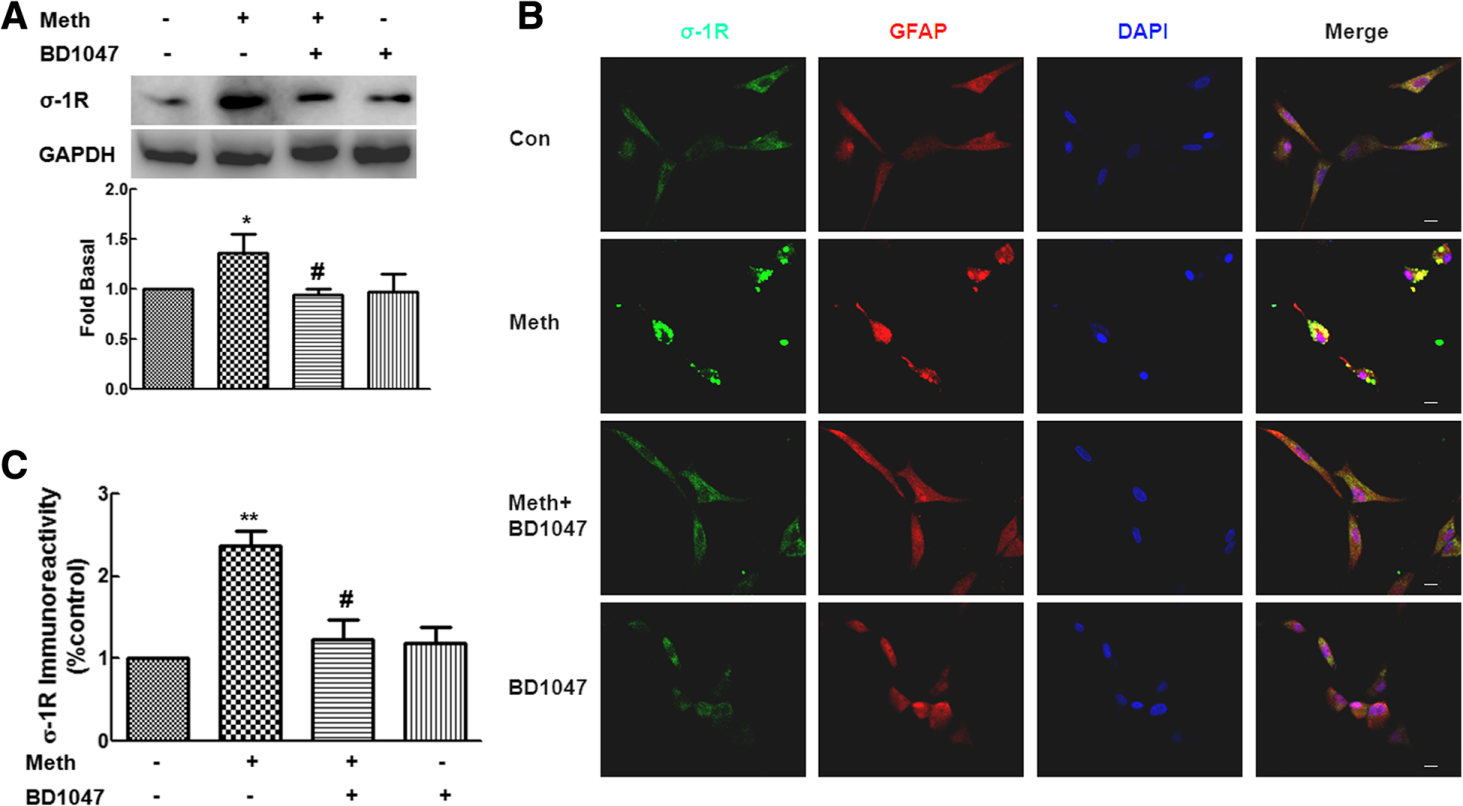
**Involvement of σ-1R in methamphetamine-mediated σ-1R expression in primary rat astrocytes. (A)** Pretreatment of primary rat astrocytes with the σ-1R antagonist-BD1047 (10 μM) inhibited methamphetamine-mediated increased expression of σ-1R. Representative immunoblots and the densitometric analysis of σ-1R/GAPDH from the three separate experiments are presented. **(B)** Immunofluorescent staining for σ-1R (green) and GFAP (red) in primary rat astrocytes treated with methamphetamine or σ-1R antagonist-BD1047 (10 μM). Scale bars all indicated 10 μm. **(C)** Intensity of σ-1R immunofluorescence was quantified in five areas of the slide using the Image J software. IOD-integrated optical density. All the data are presented as mean ± SD of three individual experiments. *P < 0.05 and **P < 0.01 versus control group; #*P* < 0.05 versus methamphetamine-treated group.

Since methamphetamine mediates signaling via binding to σ-1Rs, we next wanted to dissect the detailed mechanism(s) underlying methamphetamine-mediated enhanced expression of σ-1R. Interestingly, as shown in Figure 2A, pretreatment of primary rat astrocytes with the σ-1R antagonist-BD1047 significantly inhibited methamphetamine-mediated up-regulation of σ-1R. In addition to up-regulation of σ-1R expression, treatment of primary rat astrocytes with methamphetamine also resulted in translocation of σ-1R into the plasma membrane as evidenced by the fact that methamphetamine treatment resulted in clustering and polarization of σ-1R within the cell membrane. This effect was significantly inhibited in cells pretreated with BD1047 (Figure 2B,C). Taken together, these findings suggest that σ-1R plays a critical role in methamphetamine-mediated up-regulation of σ-1R.

### Methamphetamine-induced Src activation

**Figure 3:**
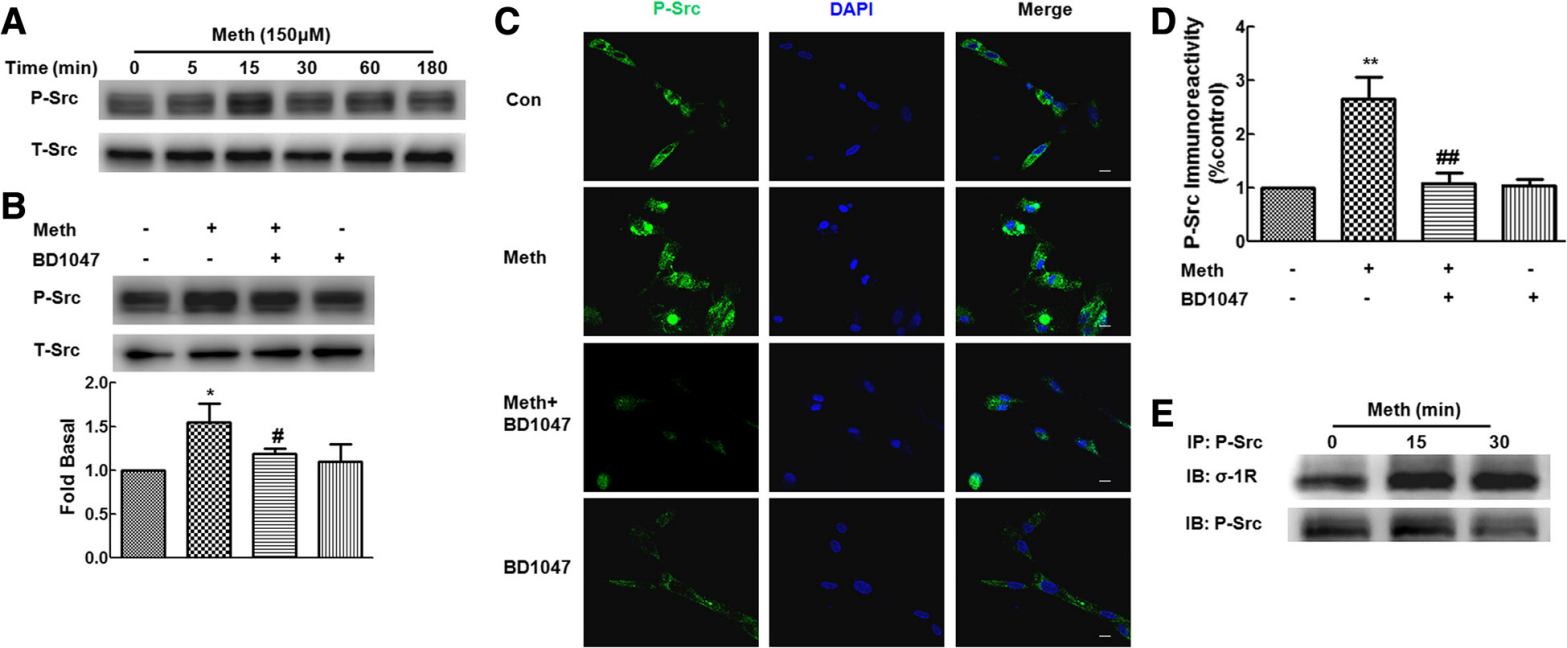
**Effect of methamphetamine on Src activation. (A)** Methamphetamine induced Src phosphorylation in a time-dependent manner in primary rat astrocytes. **(B)** Pretreatment of primary rat astrocytes with σ-1R antagonist-BD1047 (10 μM) inhibited methamphetamine-induced expression of p-Src. Representative immunoblots and the densitometric analysis of p-Src/Src from the three separate experiments are presented. **(C)** Immunofluorescent staining for p-Src (green) in primary rat astrocytes treated with methamphetamine and σ-1R antagonist-BD1047 (10 μM). Scale bars - 10 μm. **(D)** Fluorescent intensity of p-Src was quantified in five areas on the slide using the Image J software. IOD-integrated optical density. **(E)** Methamphetamine induced the interaction between σ-1R and p-Src using immunoprecipitation assay. All the data are presented as mean ± SD of three individual experiments. *P < 0.05 and **P < 0.01 versus control group; #*P* < 0.05 and ##*P* < 0.01 versus methamphetamine-treated group.

It has been shown previously that methamphetamine exposure resulted in increased expression of Src in mice [21]. We thus sought to examine whether Src also played a role in methamphetamine-mediated up-regulation of σ-1R. As shown in Figure 3A, treatment of primary rat astrocytes with methamphetamine resulted in increased phosphorylation of Src with peak response at 15 min. To further ascertain whether σ-1R was involved in methamphetamine-mediated Src activation, astrocytes were pretreated with the BD1047 for 1 h followed by incubation with methamphetamine for 15 min and then assessed for activation of Src. Pretreatment of cells with BD1047 significantly inhibited methamphetamine-mediated activation of Src (Figure 3B). Similar to increased translocation of σ-1R, phosphorylated form of Src was also significantly increased in the plasma membrane, and this was significantly inhibited in cells pretreated with BD1047 as shown in Figure 3C,D. Since both σ-1R and p-Src translocated to the cell membrane, we next wanted to examine whether there was an interaction between σ-1R and p-Src. Immunoprecipitation assay using cell lysis from methamphetamine-treated primary rat astrocytes was thus performed. As shown in Figure 3E, σ-1R-immunoreactive band was displayed in the p-Src precipitates, suggesting thereby that there was an evident interaction between σ-1R and p-Src.

### Methamphetamine-mediated activation of Src involves activation of ERK MAPK pathway

**Figure 4:**
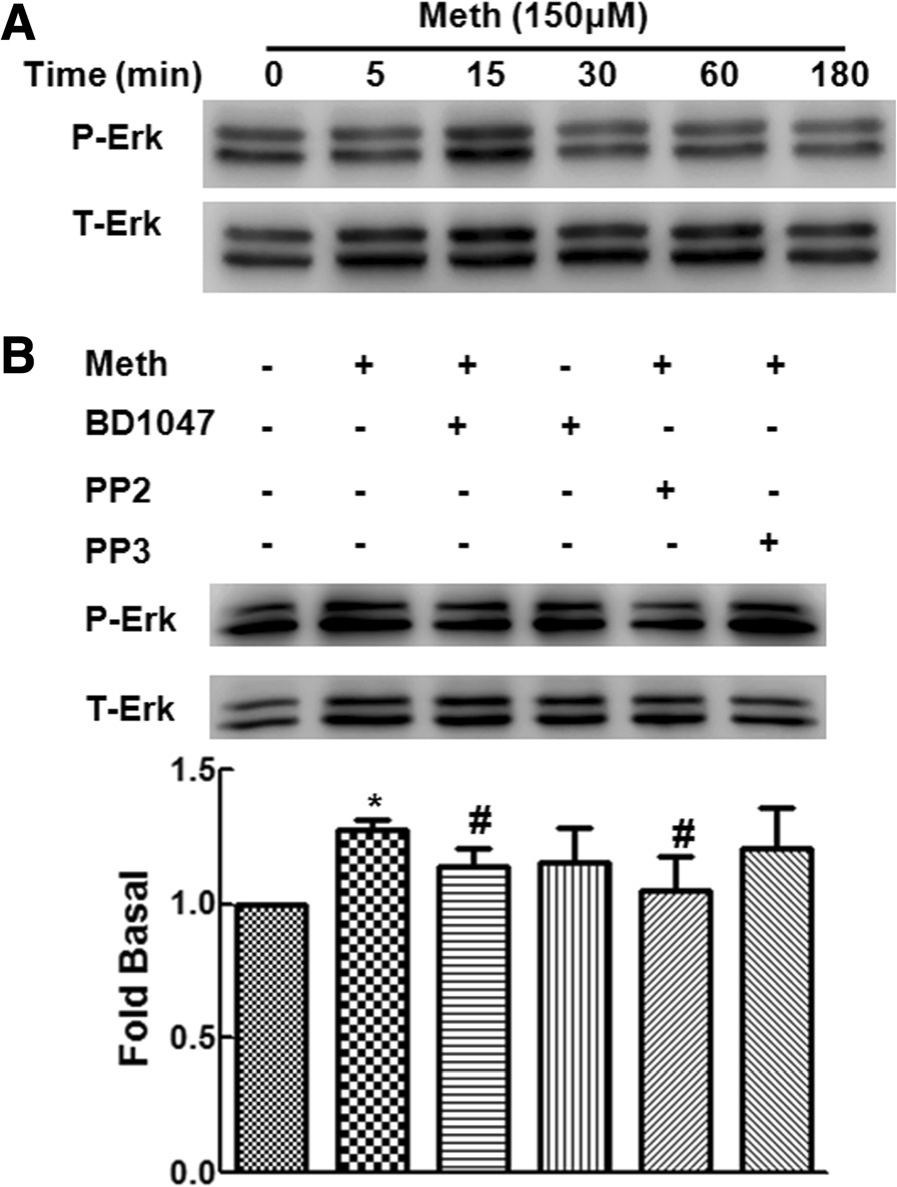
**Methamphetamine-mediated activation of Src involves activation of ERK MAPK pathway. (A)** Western blot analysis of time-dependent activation of ERK MAPKs by methamphetamine in primary rat astrocytes. **(B)** Pretreatment of primary rat astrocytes with σ-1R antagonist-BD1047 (10 μM) or Src inhibitor-PP2 (10 μM) but not its inactive analog PP3 (10 μM), resulted in inhibition of methamphetamine-mediated phosphorylation of ERK. Representative immunoblots and the densitometric analysis of p-ERK/ERK from the three separate experiments are presented. All the data are presented as mean ± SD of three individual experiments. *P < 0.05 versus control group; #*P* < 0.05 versus methamphetamine-treated group.

Since ERK MAPK kinase pathways have been shown to play critical roles in methamphetamine-mediated signaling [22-24], we next examined the involvement of ERK MAPKs in methamphetamine-mediated up-regulation of σ-1R. First, we examined the effect of methamphetamine on the activation of ERK MAPKs. As shown in Figure 4A, treatment of primary rat astrocytes with methamphetamine resulted in increased phosphorylation of ERK. We next wanted to examine the functional roles of σ-1R and Src in methamphetamine-mediated activation of ERK. Primary rat astrocytes were pretreated with inhibitors specific for the respective mediators prior to stimulation with methamphetamine and assessed for activation of ERK. As shown in Figure 4B, pretreatment of cells with σ-1R antagonist-BD1047 or the Src inhibitor-PP2 (10 μM), resulted in inhibition of methamphetamine-mediated activation of ERK. Pretreatment of cells with the inactive Src analog PP3, on the other hand, had no effect on methamphetamine-mediated activation of ERK. Together, these findings implicate the involvement of σ-1R and Src in methamphetamine-mediated activation of ERK in primary rat astrocytes.

### Methamphetamine-mediated up-regulation of σ-1R involves activation of CREB

**Figure 5:**
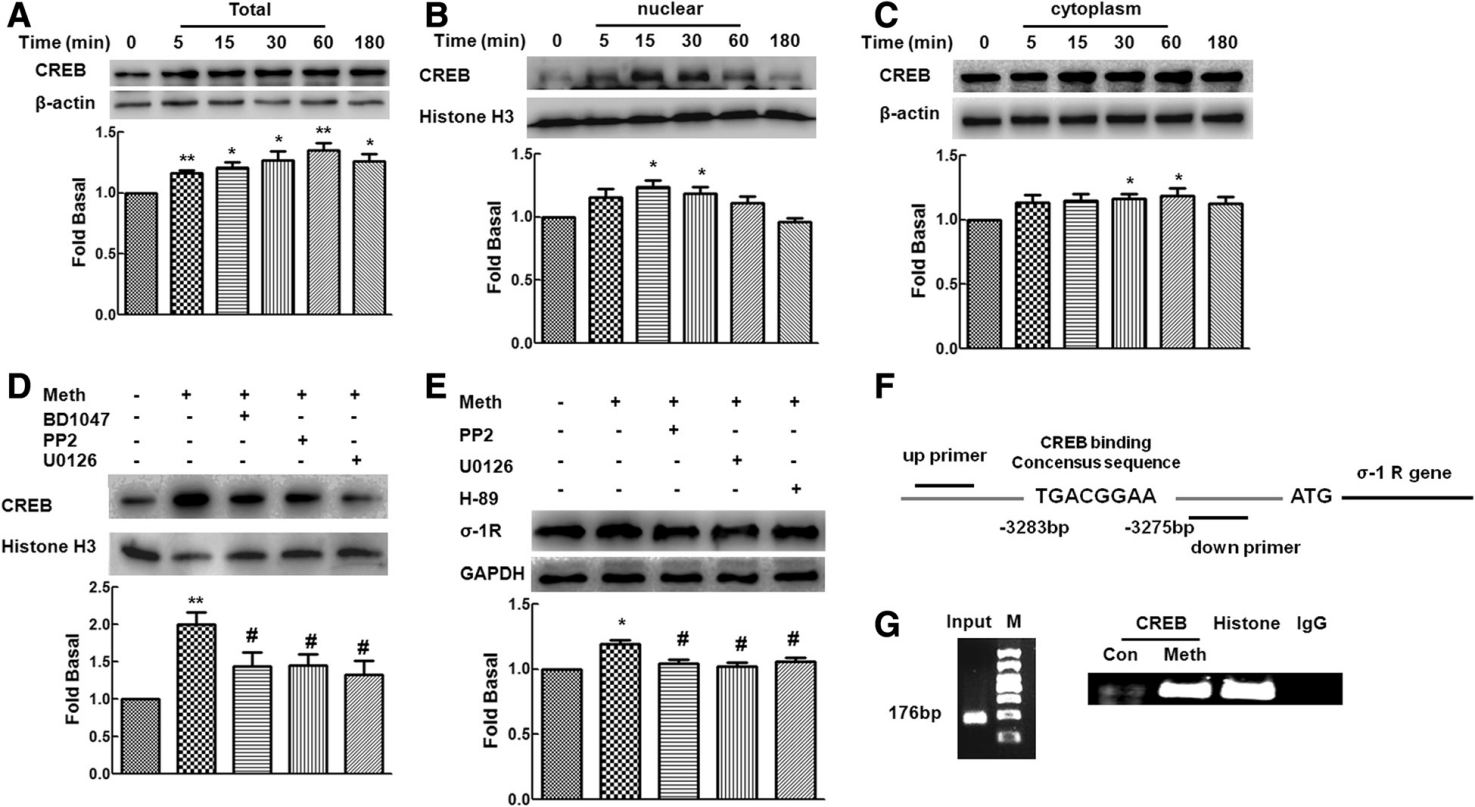
**Methamphetamine-mediated induction of σ-1R involves CREB activation. (A to C)** Effect of methamphetamine on the total level of CREB in whole cell lysates (A), nuclear (B), as well as cytoplasmic (C) fractions. **(D)** Pretreatment of astrocytes with σ-1R antagonist-BD1047 (10 μM), Src inhibitor-PP2 (10 μM), or MEK1/2 (U0126, 10 μM) significantly inhibited methamphetamine-mediated translocation of CREB into the nucleus. Representative immunoblots and the densitometric analysis of CREB/Histone H3 from three separate experiments are presented. **(E)** Pretreatment of primary rat astrocytes with Src inhibitor-PP2 (10 μM), MEK1/2 (U0126, 10 μM), or the PKA inhibitor-H89 (10 μM), resulted in inhibition of methamphetamine-mediated expression of σ-1R. Representative immunoblots and the densitometric analysis of σ-1R/GAPDH from the three separate experiments are presented. All the data are presented as mean ± SD of three individual experiments. *P < 0.05 and **P < 0.01 versus control group; #*P* < 0.05 versus methamphetamine-treated group. **(F)** Illustration of the consensus binding site of CREB to the σ-1R promoter. **(G)** ChIP assay demonstrating methamphetamine-mediated binding of CREB to the σ-1R promoter.

Mounting evidence has indicated that the signaling induced by methamphetamine leads to activation of the CREB pathway [22,25]. However, whether CREB was also involved in methamphetamine-mediated up-regulation of σ-1R remains unclear. We thus sought to examine whether CREB played a role in this process in primary rat astrocytes. Methamphetamine increased the expression of CREB in the whole cell lysates with concomitant increase in translocation of CREB into the nucleus (maximal response within 30 min) (Figure 5A,B). Methamphetamine exposure also increased the expression of CREB in the cytoplasmic fraction (Figure 5C).

The next logical step was to examine whether there existed a link that could tie together the activation of σ-1R, Src, and ERK and CREB translocation into nucleus. Primary rat astrocytes were thus pretreated with σ-1R antagonist-BD1047, Src inhibitor-PP2, or ERK inhibitor-U0126 followed by treatment with methamphetamine. As shown in Figure 5D, pretreatment with all the inhibitors resulted in inhibition of methamphetamine-mediated translocation of CREB into the nucleus.

We next wanted to examine the role of ERK, Src, and CREB in methamphetamine-mediated increased expression of σ-1R. Primary rat astrocytes were pretreated with either ERK inhibitor-U0126 or Src inhibitor-PP2 and assessed for expression of σ-1R. As shown in Figure 5E, pretreatment of cells with either of the inhibitor resulted in inhibition of methamphetamine-mediated increased expression of σ-1R. To further ascertain the role of CREB in methamphetamine-mediated expression of σ-1R, cells were pretreated with the PKA inhibitor-H89 (10 μM) and assessed for expression of sigma-1R, in response to methamphetamine. As shown in Figure 5F, pretreatment of cells with H89 resulted in inhibition of methamphetamine-mediated induction of sigma-1R, thereby implicating involvement of CREB in this process. These findings thus linked methamphetamine-mediated activation of σ-1R/Src/ERK MAPKs to downstream translocation of CREB as well as the expression of σ-1R.

As predicted from TFSEARCH (http://www.cbrc.jp/research/db/ TFSEARCH.html), there were putative CREB binding sites within the sequence upstream of the σ-1R promoter. In order to examine whether CREB physically bound to the σ-1R promoter, ChIP assays were performed. Intriguingly, treatment of primary rat astrocytes with methamphetamine resulted in increased binding of CREB to the σ-1R promoter, thereby suggesting that CREB binds to a putative regulatory element on the σ-1R promoter (Figure 5F,G).

### σ-1R-mediated activation of astrocytes induced by methamphetamine

**Figure 6:**
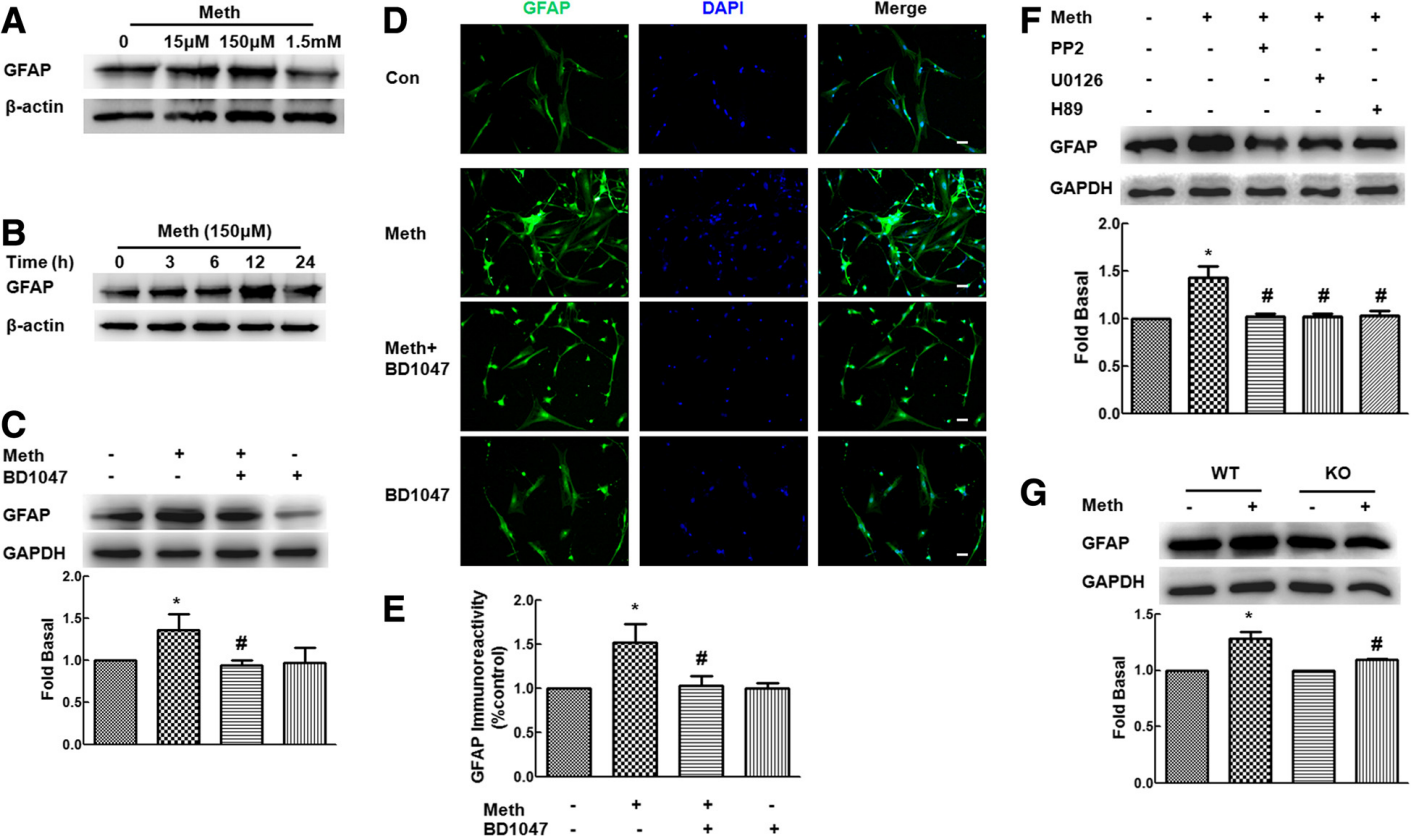
**Role of σ-1R in the activation of astrocytes mediated by methamphetamine. (A)** Exposure of primary rat astrocytes to different concentrations (15 μM, 150 μM, and 1.5 mM) of methamphetamine increased the GFAP expression with maximal response at the concentration of 150 μM. **(B)** Effect of methamphetamine (150 μM) increased the expression of GFAP in rat primary astrocytes. **(C)** Pretreatment of primary rat astrocytes with σ-1R antagonist-BD1047 significantly inhibited methamphetamine-mediated increase in GFAP expression. **(D)** Immunofluorescent staining for GFAP (green) in primary rat astrocytes treated with methamphetamine and σ-1R antagonist-BD1047 (10 μM). Scale bars - 50 μm. **(E)** Quantification of GFAP immunofluorescent intensity from five areas on the slide using the Image J software. IOD-integrated optical density. **(F)** Pretreatment of primary rat astrocytes with Src inhibitor-PP2 (10 μM), MEK1/2 (U0126, 10 μM), or PKA inhibitor-H89 (10 μM), resulted in inhibition of methamphetamine-mediated expression of GFAP. Representative immunoblots and the densitometric analysis of σ-1R/GAPDH from the three separate experiments are presented. **(G)** Compared with primary mouse astrocytes from WT mice, methamphetamine failed to increase the expression of GFAP in astrocyte isolated from σ-1R KO mice. All the data are mean ± SD of three individual experiments. *P < 0.05 versus control group; #*P* < 0.05 versus methamphetamine-treated group in astrocytes from WT mice.

Since expression of GFAP is an indication of progressive stages of astrocyte activation [26,27], we sought to examine whether the functional relevance of up-regulated σ-1R correlated with astrocyte activation. As shown in Figure 6A, astrocytes treated with varying concentrations of methamphetamine resulted in increased expression of GFAP with maximal response observed at the concentration of 150 μM methamphetamine. At this concentration of methamphetamine, there was increased expression of GFAP at 12 h (Figure 6B). To confirm the role of σ-1R in this process, activation of astrocytes was also evaluated in the presence of σ-1R antagonist-BD1047. Pretreatment of cells with BD1047 significantly inhibited methamphetamine-mediated increased expression of GFAP (Figure 6C). This finding was further confirmed by immunostaining of cells treated with methamphetamine with or without σ-1R antagonist-BD1047 with GFAP antibody (Figure 6D,E). Next, we sought to examine the functional roles of ERK, Src, and CREB in methamphetamine-mediated activation of astrocytes. As shown in Figure 6F, pretreatment of cells with each of the respective inhibitors resulted in significant inhibition of methamphetamine-mediated activation of astrocytes. In addition to the pharmacological approach, primary mouse astrocytes isolated from σ-1R KO mice were also used in this study to validate the findings. As shown in Figure 6G, compared with cells from wild-type (WT) mice, methamphetamine failed to increase the expression of GFAP in astrocytes isolated from σ-1R KO mice, thereby implicating the role of σ-1R in methamphetamine-mediated activation of astrocytes.

### Methamphetamine up-regulated σ-1R protein expression *in vivo*

**Figure 7:**
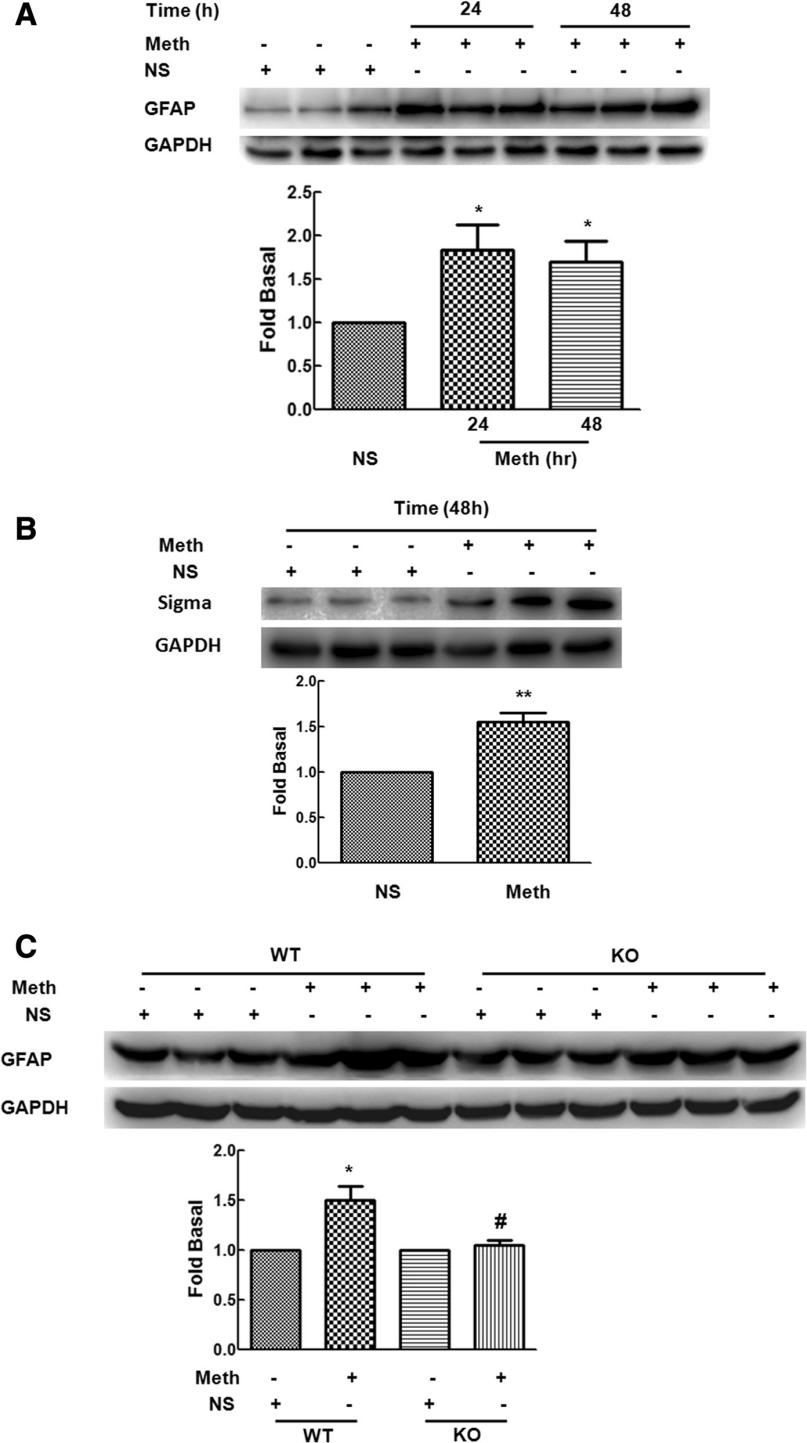
**Effect of methamphetamine on the expression of GFAP** ***in vivo*** **. (A)** Effect of methamphetamine on the expression of GFAP in striatum isolated from methamphetamine-injected mice for 24 or 48 h. **(B)** Effect of methamphetamine on the expression of σ-1R in striatum isolated from methamphetamine-treated mice for 48 h. **(C)** Administration of methamphetamine in the WT mice induced the expression of GFAP but failed to increase GFAP expression in the σ-1R KO mice. All the data are mean ± SD, *n* = 3 per group. *P < 0.05 versus control group; #*P* < 0.05 versus methamphetamine-treated group in astrocytes from WT mice.

To examine the relevance of astrocyte activation induced by methamphetamine *in vivo*, mice were injected intraperitoneally with methamphetamine (30 mg/kg body weight). As shown in Figure 7A, administration of methamphetamine induced significant astrocyte activation in the striatum as determined by the expression of GFAP at both 24 and 48 h following treatment. Moreover, 48 h after treatment, methamphetamine significantly increased the expression of σ-1R (Figure 7B). To validate the role of σ-1R in the astrocyte activation mediated by methamphetamine, we also examined the expression of GFAP in both WT and σ-1R KO mice. As shown in Figure 7C, and as expected, methamphetamine treatment failed to induce astrocyte activation in σ-1R KO mice compared with the WT mice.

## Discussion

The study presented here aimed to dissect the mechanism(s) by which methamphetamine induced the activation of astrocytes through positive-feedback mechanisms via increasing expression of itself, thus amplifying the effect of methamphetamine on astrocyte activation. While previous studies have demonstrated that methamphetamine induced both activation of astrocytes and expression of σ-1R, the molecular mechanism(s) underlying these processes are not completely understood. In the current study, we demonstrate that methamphetamine exposure of astrocytes resulted in increased expression of σ-1R as well as translocation to the cell membrane, followed by its interaction with p-Src. Downstream activation of ERK MAPK pathway and transcription factor CREB lead to positive-feedback amplification of σ-1R expression with subsequent functional activation of astrocytes. Collectively, these results for the first time demonstrate the critical role of σ-1R in methamphetamine-mediated activation of astrocytes via its own regulation. These findings thus imply σ-1R as a promising therapeutic target for amelioration of methamphetamine-mediated neurodegenerative effects.

Consistent with the previous studies, we also demonstrated that methamphetamine was able to increase the expression of σ-1R in primary rat astrocytes. Furthermore, since methamphetamine has high affinity for σ-1R [2], we investigated the role of this receptor in methamphetamine-mediated expression of σ-1R. Intriguingly, methamphetamine mediated increased expression of σ-1R via binding to σ-1R, as evidenced by the fact that pretreatment of cells with σ-1R antagonist-BD1047 abrogated increased expression of σ-1R mediated by methamphetamine.

In the CNS, σ-1R play a myriad of roles ranging from modulation of neurotransmitter release, receptor and channel regulation, calcium signaling, learning and memory, mood alterations [28], drug dependence, and even depression [29]. Intriguingly, most of the functions of σ-1R are mediated by the intricate regulation of lipids by these receptors inside the cell [30]. Our previous study demonstrated that σ-1R located in the cytoplasm was translocated into lipid raft domain of the plasma membrane in microglia treated with cocaine, which are platforms for key cellular signaling components [31]. Further study indicated that there was interaction of σ-1R and tyrosine kinase receptor-platelet-derived growth factor receptor (PDGF-R) in human brain microvascular endothelial cells [32]. However, whether σ-1R was involved in methamphetamine-mediated activation of cell signaling pathways has not yet been determined.

In agreement with our previous findings in endothelial and microglial cells [31,32], methamphetamine treatment also resulted in translocation of σ-1R from the cytoplasm to the cell membrane. Intriguingly, activation of σ-1R with methamphetamine subsequently resulted in phosphorylation of tyrosine kinase Src. Participation of Src in this process is consistent with the report demonstrating that methamphetamine treatment induces expression of Src even *in vivo* [21]. Another key feature of our findings was that methamphetamine-mediated activation of Src was dependent on activation of σ-1R. Inhibition of σ-1R activation with the antagonist-BD1047 significantly blocked methamphetamine-mediated activation of Src, suggesting thereby that σ-1R activation was upstream of Src activation mediated by methamphetamine. To our knowledge, this is the first report demonstrating a direct interaction of σ-1R with p-Src, which is consistent with the previous reports that plasma membranes serve as platform for coalescing the interaction of σ-1R with key molecules [33].

We also examined the signaling pathways involved in methamphetamine-mediated up-regulation of σ-1R. Methamphetamine induced ERK phosphorylation, which is in agreement with the effect of methamphetamine on activation of MAPKs pathway in the *in vivo* studies [22-24]. Using pharmacological approaches, our findings demonstrated the involvement of σ-1R and Src activation in methamphetamine-mediated activation of ERK. Moreover, our study also demonstrated that ERK pathway was involved in methamphetamine-mediated increased expression of σ-1R using pharmacological approach-U0126 (Figure 5E). However, contrary to our findings, Wang *et al*. reported that methamphetamine induced ERK phosphorylation, unfortunately, specific blockage ERK pathway failed to rescues methamphetamine-induced cell death of SH-SY5Y [34]. This difference could be attributable to the cell type and/or the different concentrations of methamphetamine used in the two studies.

Mounting evidence indicates that methamphetamine induces activation of CREB, a transcription factor that is downstream of ERK activation [22,35]. Consistent with these findings, our study also indicated that methamphetamine exposure induced translocation of CREB into nucleus via σ-1R with subsequent activation of Src and ERK MAPK cascade in primary rat astrocytes. Interestingly, blockage of CREB signaling significantly inhibited methamphetamine-mediated up-regulation of σ-1R (Figure 6). Since methamphetamine mediated up-regulation of σ-1R expression, we further explored the mechanism(s) underlying this process. To our knowledge, this is the first time to provide the evidence for the regulatory element containing a CREB binding site located upstream of the σ-1R promoter and identify the functional binding of CREB to the σ-1R promoter, thereby revealing a novel mechanism involving the role of CREB pathway in methamphetamine-mediated up-regulation of σ-1R in astrocytes.

**Figure 8:**
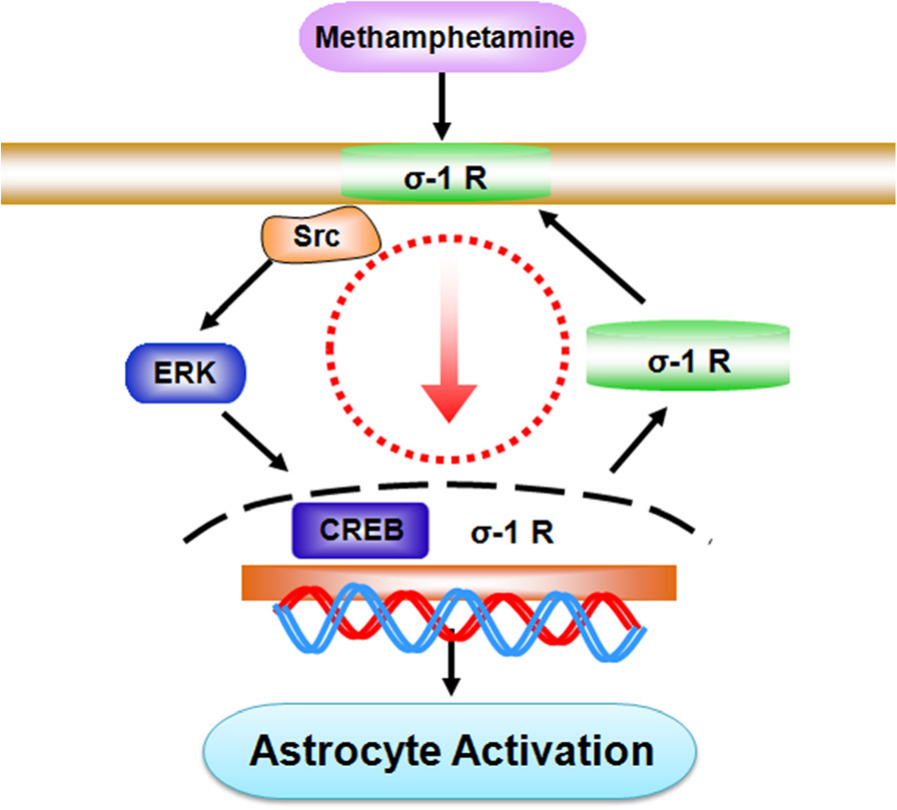
**Schematic of signaling pathways involved in methamphetamine-induced astrocyte activation via positive-feedback loop of σ-1R expression.** Exposure of astrocytes to methamphetamine leads to activation of astrocytes via up-regulation of σ-1R. Inhibition of σ-1R, Src, or ERK MAPKs resulted in the subsequent inactivation of the downstream CREB transcription factor. CREB binds to the promoter of σ-1R, leading to enhanced σ-1R expression with subsequent astrocyte activation, thereby amplifying the methamphetamine response.

It is well-recognized that astrocyte activation is a key feature of the neuroinflammatory process. Reaction of astrocytes is characterized by early proliferation and increased expression of GFAP. Our complementary *in vitro* and *in vivo* studies lend credence to methamphetamine-mediated activation of astrocytes with a concomitant increased expression of σ-1R in primary astrocytes and in striatum of methamphetamine-treated mice (Figure 8). Lack of astrocyte activation in response to methamphetamine in σ-1R KO mice further validates the role of σ-1R in this process. The significance of this study is that it is for the first time to provide the evidence that σ-1R plays a key role in methamphetamine-mediated astrocyte activation by using pharmacological as well as genetic approaches. Our study thereby indicated that σ-1R was involved in methamphetamine-mediated activation of astrocytes.

## Conclusions

In summary, our findings have outlined a detailed molecular pathway involved in methamphetamine-mediated activation of astrocytes via σ-1R with downstream activation of Src and ERK MAPK pathways, and subsequent activation of CREB resulting in increased expression of σ-1R expression, which, in turn, promotes activation of astrocytes (Figure 8). These findings have implications for activated astrocytes induced by methamphetamine. Blockage of σ-1R can be considered as an adjunct therapeutic strategy for treatment of methamphetamine-mediated neuroinflammation.

## Abbreviations

σ-1R: sigma-1 receptor
ChIP: chromatin immunoprecipitation
CNS: central nervous system
CREB: cAMP-response element-binding protein
DMEM: Dulbecco’s modified Eagle’s medium
GFAP: glial fibrillary acidic protein
KO: knockout
SD rat: Sprague-Dawley rat
TBST: Tris-buffered saline with Tween
WT: wild-type
